# Diamond particles in toothpastes: in-vitro effect on the abrasive enamel wear

**DOI:** 10.1186/s12903-022-02274-3

**Published:** 2022-06-22

**Authors:** Blend Hamza, Aralia Abdulahad, Thomas Attin, Florian J. Wegehaupt

**Affiliations:** 1 Clinic of Orthodontics and Pediatric Dentistry, Center of Dental Medicine, University of Zurich, Plattenstrasse 11, 8032 Zurich, Switzerland; 2 Clinic of Conservative and Preventive Dentistry, Center for Dental Medicine, University of Zurich, 8032 Zurich, Switzerland

**Keywords:** Diamond particles, Toothpaste, Abrasive enamel wear, Abrasives, Preventive dentistry

## Abstract

**Background:**

Diamond particles have recently been used as abrasives in toothpastes, which raises questions about its abrasive behaviour towards enamel. This study was carried out to investigate the abrasive enamel wear caused by three diamond-loaded toothpastes (Candida White Diamond: CWD, Swiss Smile Diamond Glow: SSDG, Emoform F Diamond: EFD) and to compare it with a traditional toothpaste with silica abrasive (Colgate Total Original CTO).

**Methods:**

Eighty bovine enamel samples were divided into four groups (n = 20) and brushed for 21,600 cycles (60 cycles/min) for 6 h at 2.5-N brushing force. The abrasive enamel wear was recorded with a contact profilometer. The median and interquartile range (IQR) of the abrasive enamel wear was calculated in each group. Pairwise comparisons were conducted using Wilcoxon signed rank exact test and the p value was adjusted according to Holm. Significance level was set at 0.05.

**Results:**

Diamond-loaded toothpastes caused statistically significantly higher abrasive wear than the traditional toothpaste (*p* < 0.0001). SSDG caused statistically significantly higher enamel wear (19.0 µm (11.2)) than CWD (8.4 µm (4.6)) and EFD (7.3 µm (3.9)) (*p* < 0.0001).

**Conclusions:**

Diamond-loaded toothpastes cause higher enamel wear than toothpastes with traditional abrasives and also exhibit different abrasivity behaviour compared to each other.

## Background

Brushing the teeth with a fluoridated toothpaste belongs now to our everyday routine. Beside fluoride, toothpastes are always loaded with abrasive particles to enhance the mechanical removal of the dental plaque and external stain [1]. It is important for these abrasive particles to be harder than the dental plaque (in order to remove it during brushing), but softer than the tooth hard tissue (in order to prevent its removal during brushing). Silica based particles are the most common abrasives inside toothpastes [2]. Other common abrasive particles include—but not limited to—calcium, carbonate and phosphate [3]. Due to the fact that dentine is much softer than enamel, caution was taken that the hardness of the used abrasive particles was in the range of the hardness of dentine. Toothpastes were commonly tested and controlled for their potential ability to abrade sound dentine during brushing, the so-called abrasive dentine wear [4]. In this manner, the susceptibility of enamel to such abrasive wear—the abrasive enamel wear—was gradually neglected, given the fact that it is much harder than most of the used abrasive particles [4]. However, novel toothpastes that contain diamond abrasive particles were recently introduced to the market [5, 6]. This started raising questions about the safety of such toothpastes in means of its potential abrasivity on both dentine and enamel.

A recent study investigated the relative enamel and dentine abrasivity (REA and RDA) of three toothpastes with diamond particles [7]. The study reported that while these toothpastes behaved quite mildly on dentine surface (RDA = 12, 14 and 42), they were extremely abrasive on enamel surface (REA = 51, 177 and 244). To understand the gravity of these numbers, it should be mentioned that the International Standard Organisation (ISO 11609:2017) stated that an REA of a toothpastes should not exceed the value of 10. Nevertheless, the used abrasivity testing methods (RDA and REA) are relative and known for producing fluctuating values (up to 20%) [4]. It is therefore plausible to investigate the absolute abrasive wear caused by such toothpastes using other standardised methods, namely profilometry. This study was carried out to investigate and compare the abrasive enamel wear caused by three toothpastes utilising diamond abrasive particles “diamond-loaded toothpaste” (Candida White Diamond (REA = 244 ± 76), Swiss Smile Diamond Glow (REA = 177 ± 70), Emoform F Diamond (REA = 51 ± 25)) and a toothpaste utilising—traditional—silica abrasive (Colgate Total Original (REA = 4 ± 2)). Since there is a controversy in the literature whether the profilometric method of measuring abrasive wear produces similar results/rankings as the radiotracer method [4, 8, 9], and since the abrasivity of the tested toothpastes in the present study had already been measured using the radiotracer method in a previous study [7], the results of the present study could contribute to the current knowledge about the discrepancies between both methods. The null hypothesis was that there would be no difference in the abrasive enamel wear between diamond-loaded and traditional toothpaste nor between the tested diamond-loaded toothpastes.

## Materials and methods

**Fig. 1:**
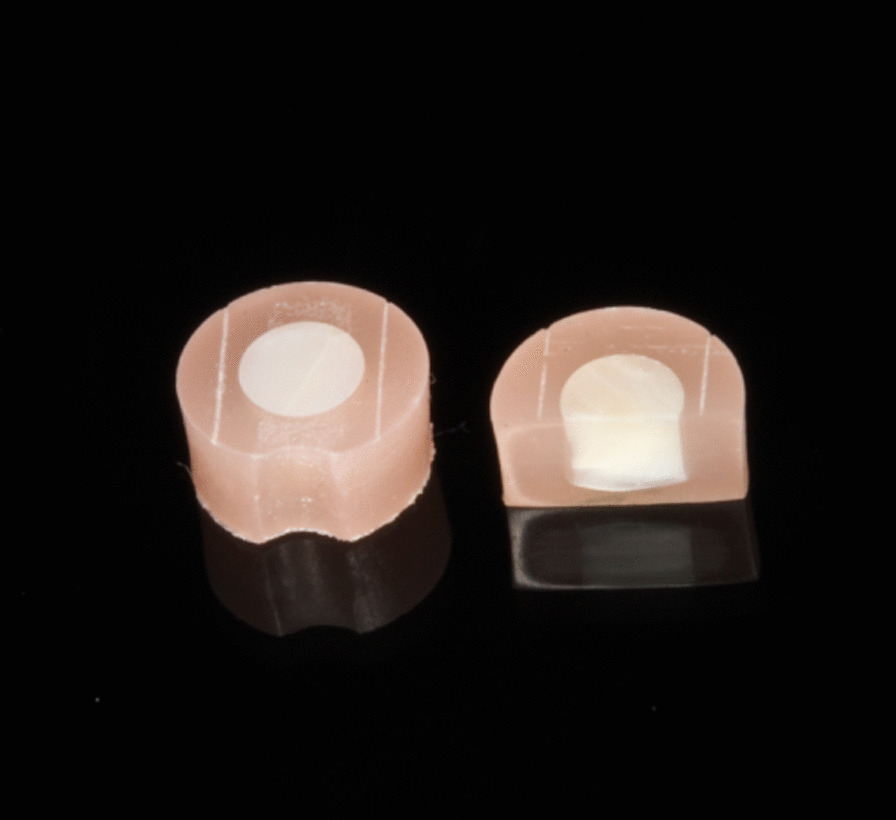
Two enamel samples. The sample on the right was sectioned for better visualisation

Eighty enamel samples were prepared from 20 extracted permanent bovine incisors for this study. The bovine sacrifice was carried out solely for food processing and had no relation with the present study. Four samples were milled out from the crown of each incisor using a trephine mill with an inner diameter of 3 mm. The samples were then embedded in acrylic resin (Paladur, Heraeus Kulzer, Hanau, Germany), which was polymerised inside a laboratory incubator (Palamet elite, Heraeus Kulzer) at 45 °C and 2 bar for 10 min. The samples were ground in a grinding machine (Struers Tegramin-30, Erkrath, Germany) using 1200-, 2000-, and 4000-grit silicon-carbide papers for 10, 20 and 30 s, respectively under constant water cooling. Using a sharp metal pen held in a custom-made device, two parallel lines were scraped into the samples’ surface. These parallel lines were made in the embedding material as near to the enamel surface as possible and were used later as reference lines for the profilometric analysis. Parts of the enamel on the sample sides were covered using an adhesive tape to protect the area beneath from abrasion and aid the profilometric recording (Fig. 1). The baseline profilometric recording was carried out for all samples using a contact profilometer (Perthometer S2, Mahr, Göttingen, Germany) according to a previously established protocol [10]. Five parallel profiles (distance = 250 µm, recording accuracy = 40 nm) were recorded for each sample. Using a prefabricated jig, the exact positioning and repositioning of the samples in the profilometer was ensured.

**Fig. 2:**
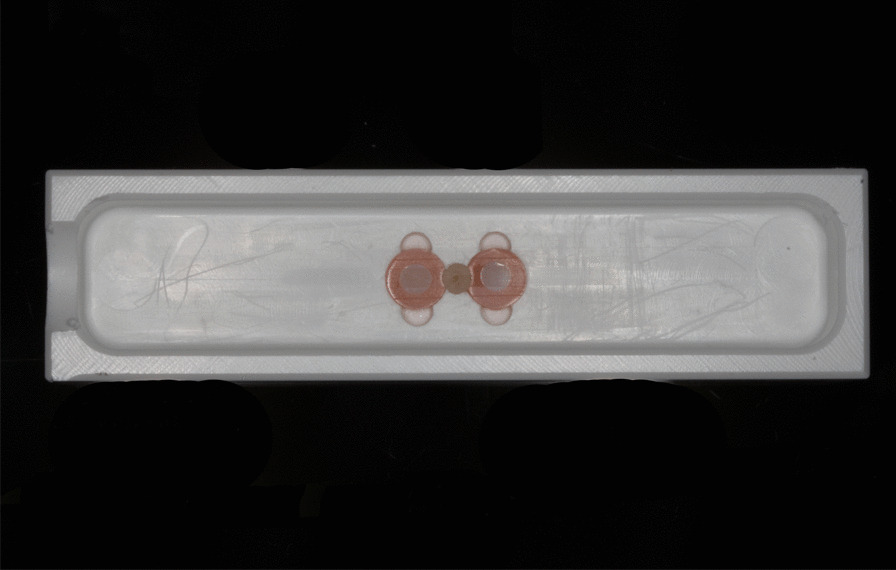
The brushing container, in which the samples were covered with the respective toothpaste slurry and subjected to the brushing sequence

Thereafter, samples were divided into four groups based on tested toothpaste. Each group had 20 samples milled out from 20 different incisors. In other words, each group had one sample from the same incisor to ensure a certain sample homogeneity throughout the four groups. Group 1 was brushed using a slurry of Colgate Total Original (CTO; Colgate-Palmolive, Poland), group 2 using a slurry of Candida White Diamond (CWD; Mibelle AG, Switzerland), group 3 using a slurry of Swiss Smile Diamond Glow (SSDG; Curaden AG, Switzerland), and group 4 with Emoform F Diamond (EFD; Dr. Wild & Co. AG, Switzerland). The brushing sequence was carried out in a custom-made 6-place-cross-brushing-machine, where brushing containers (with two samples each, Fig. 2) filled with the respective slurry were fixed tight. The samples were brushed with a medium-bristles standard toothbrush (Paro M43, Esro AG, Thalwil, Switzerland) for 6 h, at 2.5-N brushing force and 60 cycles/min brushing speed. With 1.5-h intervals, the slurries inside the brushing containers (3 ml) were replaced with fresh ones. The slurries were prepared by mixing the respective toothpaste with artificial saliva [11] at 1:2 ratio. After the brushing sequence (6 h), the samples were thoroughly rinsed with tap water and final profiles were recorded. Table 1 summarises the study design and Table 2 shows the composition of the tested toothpastes according to the manufacturer.

**Table 1:** Study design

Preparation of 80 bovine enamel samples from 20 teeth (samples A–D per tooth)			
Recording baseline profiles			
Group 1 n = 20 (samples A)	Group 2 n = 20 (samples B)	Group 3 n = 20 (samples C)	Group 4 n = 20 (samples D)
Brushing sequence (Bristle stiffness = medium, Paro M43) (60 cycles/min for total 21,600 brushing cycles; 250 g, Slurry 1:2, fresh slurry after each 5400 brushing cycles)			
Colgate total original REA: 4 ± 2 n = 20	Candida white diamond REA: 244 ± 76 n = 20	Swiss Smile diamond glow REA: 177 ± 70 n = 20	Emoform F diamond REA: 51 ± 25 n = 20
Recording of final profiles			
Calculating the resulting abrasive enamel wear (in µm)

**Table 2:** The composition the tested toothpastes according to the manufacturer

Tested toothpastes (manufacturer)	Composition	Utilised abrasives
Candida White Diamond *(Mibelle AG, Switzerland)*	Aqua, Hydrogenated starch hydrolysate, Potassium citrate, Sodium lauryl sulfate, Xantham gum, Aroma, Sodium acrylates, Sodium Saccharin, Zinc chloride, Methylparaben, Allantoin, Limonene, Linalool, Benzyl alcohol Sodium fluoride (1450 ppm)	Diamond Powder Hydrated silica
Emoform F Diamond *(Dr. Wild & Co. AG, Switzerland)*	Aqua, Glycerin, Sorbitol, Propylene glycol, Xylitol, PEG-8, PEG-40-Hydrogenated castor oil, Cocamidopropyl betaine, Cellulose, Gum, Potassium phosphate, Aroma, Sodium chloride, Rebaudioside A, Limonene, CI 42090, Sodium fluoride (1400 ppm)	Diamond Powder Silica
Swiss Smile Diamond Glow *(Curaden AG, Switzerland)*	Aqua, Glycerin, Sorbitol, Titanium dioxide, Bentonite, Decyl glucoside, Mica, Tocopherol, Glucose Oxidase, Aroma, Cocamidopropyl betaine, Titanium dioxide, Xanthan Gum, Potassium chloride, Sodium saccharin, Curcuma xanthorrhiza root extract, Aloe barbadensis juice extract, Sodium hydroxide, Sodium benzoate, Maltodextrin, Tin oxide, D-limonene, linalool, Eugenol Sodium Monofluorophosphate (980 ppm)	Diamond Powder Hydrated silica Hydroxyapatite Silica
Colgate Total Original *(Colgate-Palmolive, Poland)*	Aqua, Glycerin, PVM/MA Copolymer, Sodium lauryl sulfate, Cellulose, Gum, Aroma, Sodium hydroxide, Carrageenan, Triclosan, Sodium saccharin, Limonene, CI 77891, Sodium fluoride (1450 ppm)	Hydrated silica

As neither the amount of the particles nor the particle size of the utilised abrasives is declared by the manufacturers, an attempt to visualise the abrasives in each of the tested toothpastes was undertaken using scanning electron microscopy (SEM). The respective slurry was gradually washed out with distilled water and a drop of it was applied on a polycarbonate SEM holder [5].

### Statistical analysis

Median and interquartile range (IQR) of the abrasive enamel wear for each tested group were calculated. The Shapiro–Wilk test indicated a significant departure from the normality (*p* < 0.001). Pairwise comparisons between the groups were conducted using Wilcoxon signed rank exact test and the *p* value was adjusted according to Holm. Significance level was set at 0.05. Data was processed with R software (The R Foundation for Statistical Computing; Vienna, Austria; www.R-project.org).

## Results

SSDG caused the highest abrasive enamel wear (19.0 µm (11.2)). This abrasive wear was statistically significantly higher than all other groups (*p* < 0.0001). CWD and EFD caused the second and the third highest abrasive wear with (8.4 µm (4.6)) and (7.3 µm (3.9)), respectively and were not statistically significantly different (*p* = 0.1). All diamond-loaded toothpastes caused statistically significantly higher enamel wear than the traditional toothpaste CTO, which caused (0.1 µm (0.1)) abrasive enamel wear (*p* < 0.0001). Table 3 shows the abrasive enamel wear for each group.

**Table 3:** Abrasive enamel wear (µm) in the experimental groups

Group	Median	IQR	Minimum	Maximum
Colgate total original	0.11 (A)	0.12	0.00	0.74
Candida white diamond	8.41 (B)	4.60	4.23	16.93
Emoform F diamond	7.33 (B)	3.97	3.90	17.53
Swiss smile diamond glow	19.00 (C)	11.20	8.61	42.55

Same letters after the median indicate no statistically significant difference between the groups

**Fig. 3:**
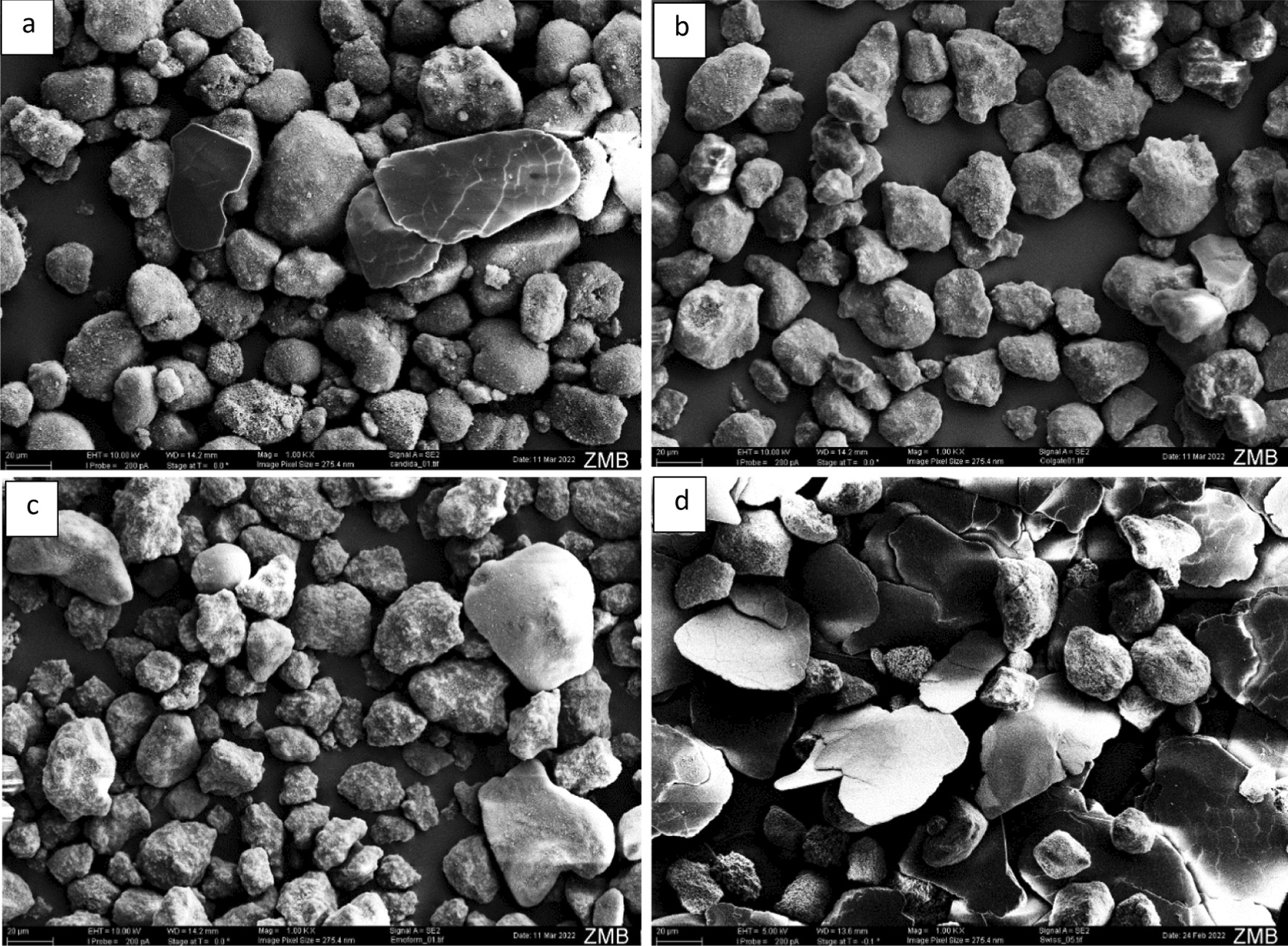
SEM photos (× 1000, scale = 20 µm) visualising the abrasives used in the tested toothpastes. Candida White Diamond (**a**), Colgate Total Original (**b**), Emoform F Diamond (**c**), Swiss Smile Diamond Glow (**d**)

Figure 3 depicts the obtained SEM photos of the utilised abrasives in each tested toothpaste. It is not possible to characterise the nature of the captured abrasives (diamond vs. silica) depending only on SEM photos. However, an idea of the particles’ size and shape could be obtained. It could be observed that SSDG abrasives tend to have more sharp edges compared to all other toothpastes.

## Discussion

Diamond particles have found their way as abrasives into some novel toothpastes. This has raised some concerns regarding the abrasive effect of those particles on dental enamel. This study was therefore conducted to investigate the abrasive enamel wear caused by three novel toothpastes with diamond particles. The results show that diamond-loaded toothpastes cause higher abrasive enamel wear than traditional toothpastes and also show differences in the abrasivity behaviour between the diamond-loaded toothpastes. The null hypothesis was therefore rejected.

Eighty enamel samples were prepared from bovine teeth for this study. Bovine enamel has similar physical and chemical properties as human enamel, and is considered a suitable alternative in abrasion studies [12, 13]. Furthermore, the large surfaces of bovine crowns allow the preparation of several samples from one tooth. Although bovine enamel was found to abrade similarly as human enamel [12], some studies reported that both behave differently under other situations (e.g., when subjected to erosion) [14–16]. Therefore, it should be mentioned that bovine enamel might not function as a like-for-like replacement for human enamel in every situation. The samples were brushed for 21,600 cycles (60 cycles/min for 6 h). Taking the clinical recommendation to brush teeth for 2 min, this corresponds to 4-year clinical brushing time, when a patient brushes her/his teeth three times a day [17]. The brushing force, at which the samples were brushed (2.5 N), lies within the brushing force applied by most of the abrasion studies (2–3 N) [18].

All tested toothpastes with diamond particles resulted in a statistically significantly higher abrasive enamel wear than the traditional toothpaste. This finding corresponds to the higher REA values also measured for these toothpastes and is attributed to the higher hardness of the diamond particles compared to dental enamel [7].

However, the fact that SSDG caused statistically significantly higher enamel wear than the other two toothpastes with diamond particles and the fact that CWD and EFD caused statistically comparable enamel wear in this study cannot be supported by their recently measured REA values. In the study of Hamza et al. [7] CWD had the highest REA value (244 ± 76) followed by SSDG (177 ± 70) and EFD (51 ± 25). On one side, some discrepancy between the radiotracer and the here used profilometry method has already been reported [4, 8], which is corroborated by the findings of the present study. On the other hand, it could also be argued that the manufacturers might have altered the amount and/or the properties of the diamond particles utilised in their toothpastes during the last two years leading to the current—different—abrasive behaviour. Therefore, the higher abrasive wear caused by SSDG could be attributed to more incorporated diamond particles or different diamond particle sizes. Furthermore, the abovementioned sharp edges observed in the abrasives utilised in SSDG might have also contributed to this observation [19]. The fact that SSDG utilises hydroxyapatite would rather not explain its higher abrasive behaviour compared to the other diamond toothpastes as the latter is a medium-hard abrasive and softer than enamel [2].

In general, it is safe to assume that the profilometric method of measuring a toothpaste’s abrasivity is much simpler than the radiotracer method (REA/RDA). It does not involve any neutron bombardment of the samples, and thus could be considered environment-friendlier [20]. Furthermore, the profilometric method has the advantage of directly measuring the abrasive wear compared to the indirect—relative—measurement provided by the radiotracer method [4]. However, González-Cabezas et al. [4] stated that the radiotracer method offers lower variations than the profilometric method (i.e., is more robust) and that the latter still needs to be better developed and refined.

Based on the present results, the diamond toothpaste with the highest enamel abrasivity (SSDG) would cause 4.75 µm enamel loss in one year. The time needed to cause 1 mm enamel loss would hence be more than 200 years. This seems safe (in terms of not completely abrading enamel and exposing dentine in a lifetime) also when taking the relatively thin enamel layer (≤ 1 mm) at the cervical third of human tooth crowns into consideration [21, 22]. However, other factors related to toothbrushing, which could increase the abrasive wear (e.g., the stiffness of the used toothbrush, the applied brushing force, using a sonic toothbrush) should be kept in mind. Another factor to be considered is the mineral quality of the enamel being brushed. Wegehaupt et al. [6] reported that a diamond-containing toothpaste (CWD) caused higher abrasive enamel wear when the enamel was previously eroded compared to sound enamel.. Regardless, a toothpaste abrasivity should always be kept in line with the cleaning efficacy it offers. A recent study reported similar cleaning efficacy of a diamond toothpaste to traditional toothpastes [23]. In this regard, other factors (e.g., the toothpaste abrasivity towards dentine, effect on the surface gloss and roughness of enamel and composite restorations) should also be kept in mind when advising patients. For instance, a recent study reported that EFD presented better gloss values than CWD when both toothpastes were used to brush composite restorations in vitro [24]. The authors also attributed the finding to possible differences in diamond particle size and concentration.

One of the limitations of the present study is that the sample size was not calculated, which presents a limitation in interpreting the results [25]. Furthermore, the present study only investigated the abrasive enamel wear caused by diamond-containing toothpastes using a medium-bristles toothbrush. Future studies should consider investigating dental plaque cleaning efficacy and the surface roughness offered by diamond-containing toothpastes in comparison to traditional toothpastes. Investigating the interplay between factors that can influence the abrasive enamel wear (e.g., stiffness of the toothbrush, brushing forces, other toothpaste active ingredient, etc.) would also be beneficial.

## Conclusions

Based on this in-vitro study and within its limitations, it could be concluded that toothpastes utilising diamond abrasive particles cause higher enamel wear than toothpastes with traditional abrasives and also exhibit different abrasivity behaviour compared to each other.

## Data Availability

The datasets generated and/or analysed during the current study are available in the [Zenodo] repository, [https://doi.org/10.5281/zenodo.6390456].

## Abbreviations

REA: Relative Enamel Abrasivity
SSDG: Swiss Smile Diamond Glow toothpaste
EFD: Emoform F Diamond toothpastes
CWD: Candida White Diamond toothpaste
CTO: Colgate Total Original toothpaste
